# Complete genome sequence of *Oscillospiraceae* bacterium strain MB08-C2-2, isolated from the feces of the black soldier fly larvae

**DOI:** 10.1128/mra.00792-25

**Published:** 2025-11-06

**Authors:** Song Wang, Jingjing Zhao, Yin Li, Chih-Hung Wu, Guowen Dong, Wangchuan Xiao, Chao-Jen Shih, Yen-Chi Wu, Wei-Ling Zhang, Lintao Wu, Wanling Qiu, Hangying Zhang, Luyao Liu, Mengyan Han, Wenshen Lin, Sheng-Chung Chen

**Affiliations:** 1 School of Chemistry and Materials, Fujian Normal University 12425, Fuzhou, Fujian, People’s Republic of China; 2 School of Resources and Chemical Engineering, Sanming University 66283, Sanming City, Fujian, People’s Republic of China; 3 Fujian Provincial Key Laboratory of Resources and Environmental Monitoring and Sustainable Management and Utilization, Sanming University 66283, Sanming, Fujian, People’s Republic of China; 4 College of Environment and Safety Engineering, Fuzhou University 12423, Fuzhou, Fujian, People’s Republic of China; 5 Bioresource Collection and Research Center, Food Industry Research and Development Institute, Hsinchu, Taiwan, People’s Republic of China; 6 School of Resources and Environment, Fujian Agriculture and Forestry University 12449, Fuzhou, Fujian, People’s Republic of China; Indiana University, Bloomington, Indiana, USA

**Keywords:** *Oscillospiraceae* bacterium, black soldier fly, gut microbiota

## Abstract

Here, we report the complete genome sequence of *Oscillospiraceae* bacterium MB08-C2-2 (=BCRC 81394), isolated from feces of black soldier fly (*Hermetia illucens*) larvae, comprising 3,292,498 bp, a GC content of 49.67%, and identification of a 72,574 bp plasmid for further species delineation and comparative genomic analysis.

## ANNOUNCEMENT

**Fig 1:**
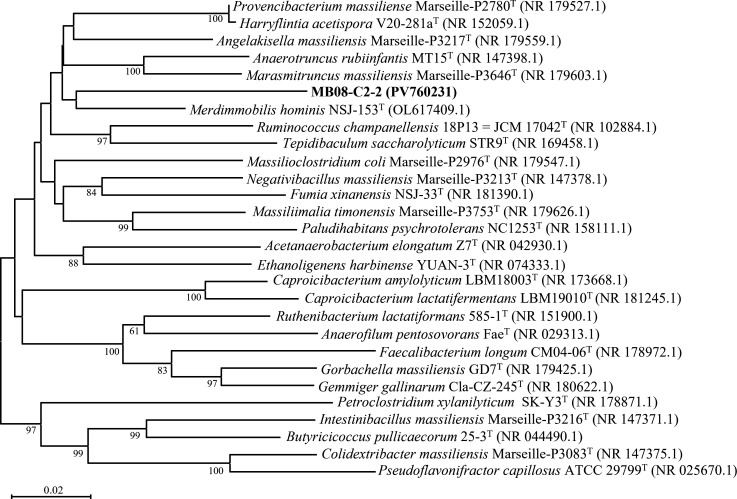
Maximum likelihood tree based on 16S rRNA gene sequences of strain MB08-C2-2 and related taxa. Sequence alignment was performed using Clustal W (9), and the Tamura–Nei model (10) was applied as the nucleotide substitution model. Bar, 0.02 substitutions per nucleotide position. Bootstrap values are expressed as percentages of 1,000 replicates.

Strain MB08-C2-2 was isolated from the feces of *Hermetia illucens* (black soldier fly, BSF) larvae, which are widely used in organic waste recycling and as a protein source (1). BSF eggs purchased from Taobao in June 2020 were reared at ~25°C for several generations; each generation (~38–45 days) involved transferring about 150–200 newly hatched larvae to new container. The batch used was estimated to be in the seventh or eighth generation. For enrichment of anaerobic bacteria, mixed feces were collected on May 30, 2021, using a sterile spoon and inoculated into methanogen broth with tungsten (MB/W) medium (per liter: 1 g MgCl_2_·7H_2_O, 0.5 g KCl, 0.1 g CaCl_2_·2H_2_O, 0.4 g K_2_HPO_4_, 1 g NH_4_Cl, 10 mL trace element solution, 2 g yeast extract, 2 g tryptone, 0.5 mL Na-resazurin solution (0.1%), 4 g NaHCO_3_, vitamin solution, 0.25 g L-cysteine-HCl·H_2_O, 0.25 g Na_2_S·9H_2_O, headspace: N_2_:CO_2 _= 4:1) (2, 3) and incubated at 25°C for 1 month. The strain was isolated by serial dilution and the rolling-tube technique (4) on MB/W medium at 25°C. The identity of the isolate was confirmed by 16S rRNA gene clone sequencing using 8F/1492RU primers (5). Purity was confirmed by morphological observation, 16S rRNA gene analysis, and genome sequencing. Based on EZBioCloud 16S-ID analysis (6), strain MB08-C2-2 (PV760231) showed the highest similarity (91.76%) to *Merdimmobilis hominis* NSJ-153^T^ (7), and the 16S rRNA gene-based phylogenetic tree constructed by MEGAX (8) indicated a distinct lineage within the family *Oscillospiraceae* (Fig. 1).

Strain MB08-C2-2 was deposited in the Bioresource Collection and Research Center (BCRC 81394), Taiwan. It was cultivated in MB/W medium at 30°C, and genomic DNA was extracted using a modified method of Johnson (11) and Jarrell et al. (12). Briefly, cells from a 500 mL culture were lysed with 1% SDS, and DNA was purified by phenol-chloroform extraction and ethanol precipitation, then quantified by UV–Vis spectrophotometry.

Whole-genome sequencing was performed using the Illumina Novaseq 6000 platform and the Oxford Nanopore MinION (Guangdong Magigene). For Illumina sequencing, libraries were prepared with the ALFA-SEQ DNA Library Prep Kit (FINDROP, Guangzhou), assessed by Qubit 4.0 Fluorometer (Life Technologies) and Qsep400 (Houze Biological Technology), and sequenced to produce 150 bp paired-end reads. Raw reads were processed with fastp v0.23.4 (13) to remove adapters, trim low-quality bases, and discard short fragments, yielding 10,921,060 clean reads (Table 1).

**TABLE 1:** Genome characteristics of *Oscillospiraceae* bacterium strain MB08-C2-2

Characteristics	Value
Sequencing and assembly features	
No. of Illumina clean reads	10,921,060
No. of Nanopore clean reads	492,714
Total no. of Illumina bases	1,631,923,742 bp
Total no. of Nanopore bases	1,735,415,527 bp
Genome coverage (Illumina; Nanopore)	496×; 516×
Genome features	
Chromosome size (GC content)	3,219,924 bp (49.67%)
Plasmid size (GC content)	72,574 bp (45.58%)
Total no. of genes	2,994
No. of coding sequences	2,929
No. of pseudogenes	29
No. of rRNAs	9
No. of tRNAs	52
No. of noncoding RNAs	4

For Nanopore sequencing, size-selected DNA (>1 Kb) was processed using the SQK-LSK109 kit and sequenced on an R9.4.1 flow cell. Base calling used Guppy v.6.5.7 (high-accuracy model), and reads were filtered with NanoFilt v2.8.0 (14), yielding 492,714 reads (*N*_50 _= 6,634 bp; average = 3,522 bp; total = 1,735,415,527 bp; Table 1).

Hybrid *de novo* assembly was performed with Unicycler v0.5.0 (15), which identified overlaps, trimmed ends, and confirmed circular topology (without genome rotation). The genome totals 3,292,498 bp, comprising one circular chromosome (3,219,924 bp; 49.67% GC) and one circular plasmid (72,574 bp; 45.58% GC) (Table 1). The average short-read and long-read coverages were 496× and 516×, respectively. The genome assembly was annotated upon its deposit to NCBI via PGAP v6.6 (16). Default parameters were used for all software tools.

## Data Availability

Both Illumina and Nanopore raw reads have been deposited in NCBI and are available through the SRA database, accession numbers SRR34574864 and SRR34574863, respectively. Assemblies have been deposited in GenBank under the accession numbers CP141729.1 (chromosome), and CP141730.1 (plasmid). The version of the genome described in this paper is the first version. The BioProject and BioSample accession numbers are PRJNA1053915 and SAMN38879520, respectively.
